# Usage evaluation of the Physiotherapy Evidence Database (PEDro) among Brazilian physical therapists

**DOI:** 10.1590/bjpt-rbf.2014.0104

**Published:** 2015-09-01

**Authors:** Mark R. Elkins, Anne M. Moseley, Rafael Z. Pinto

**Affiliations:** 1 Department of Respiratory Medicine, Royal Prince Alfred Hospital, Sydney, Australia; 2 The George Institute for Global Health, Sydney Medical School, University of Sydney, Sydney, Australia; 3 Departamento de Fisioterapia, Faculdade de Ciências e Tecnologia, Universidade Estadual Paulista (UNESP), Presidente Prudente, SP, Brazil

**Keywords:** evidence-based practice, rehabilitation, physical therapy

## Abstract

**BACKGROUND:**

It is unclear whether the Physiotherapy Evidence Database (PEDro) is widely and equally used by physical therapists in Brazil. As PEDro is considered a key resource to support evidence-based physical therapy, analyses of PEDro usage could reflect the extent of dissemination of evidence-based practice.

**OBJECTIVE:**

To describe the usage of PEDro among the five regions of the World Confederation for Physical Therapy (WCPT) and, in more detail, in the South American region and Brazil over a 5-year period.

**METHOD:**

PEDro home-page sessions and the number of searches performed were logged for a 5-year period (2010-2014). Absolute usage and relative usage were calculated for each region of the WCPT, each country in the South American region of WCPT, and each Regional Council (CREFITO) in Brazil.

**RESULTS:**

Europe had the highest absolute and relative usage among the five regions of the WCPT (971 searches per million-population per year), with the South American region ranked 4th in absolute terms and 3rd in relative terms (486). Within the South American region, Brazil accounted for nearly 60% of searches (755). Analysis at a national level revealed that usage per physical therapist in Brazil is very low across all CREFITOs. The highest usage occurred in CREFITO 6 with 1.3 searches per physical therapist per year.

**CONCLUSIONS:**

PEDro is not widely and equally used throughout Brazil. Strategies to promote PEDro and to make PEDro more accessible to physical therapists speaking Portuguese are needed.

## Introduction

The evidence-based practice movement has gained ground steadily among physical therapists worldwide over the past decade^1^. Evidence-based physical therapy has been defined as the integration of relevant, high-quality research with practice knowledge and patient preferences^2^. To effectively translate evidence into practice, physical therapists must be able to (i) convert information needs into answerable clinical questions, (ii) track down the best evidence to answer the question(s), (iii) critically appraise the evidence for its validity and applicability, (iv) integrate the evidence with clinical expertise and with patients' unique biologies, values and circumstances, and, finally, (v) evaluate his/her effectiveness and efficiency in executing steps (i) to (iv) and seek ways to improve them in the future^2^^,^ 3. Importantly, there is growing evidence to suggest that physical therapists have positive attitudes towards evidence-based practice and often report that quality of patient care is better when evidence is used^4^.

Common obstacles to the use of evidence-based practice by physical therapists include time constraints, the perception that there is limited high-quality research available, and inadequate critical appraisal skills^4^. One of the most common information needs in physical therapy practice relates to questions about the effects of interventions. These clinical questions are best answered by evidence-based clinical practice guidelines, systematic reviews of randomized clinical trials, or reports of well-conducted randomized controlled trials^5^. The Physiotherapy Evidence Database (PEDro; http://www.pedro.org.au) was developed to help physical therapists overcome some of the obstacles to implementing an evidence-based approach to intervention^6^. While it is widely acknowledged that existing evidence does not answer *all* important clinical questions in physical therapy, with more than 23,500 randomized controlled trials, 5,200 systematic reviews and 513 evidence-based clinical practice guidelines indexed in PEDro^7^, PEDro contains evidence to answer many clinical questions. PEDro is an important resource for physical therapists to use to find high-quality research to answer clinical questions about the effects of interventions because PEDro is one of the most comprehensive databases indexing reports of randomized controlled trials of physical therapy interventions^8^^,^ 9. Unlike other databases, which index a wide range of healthcare research (e.g., Lilacs, The Cochrane Library, and PubMed), PEDro only indexes evidence about the effects of physical therapy interventions and ranks search results by method (guidelines, reviews, then trials) and, for trial reports, ratings of methodological quality and the completeness of statistical reporting. This ranking of search results may assist physical therapists to identify high-quality research to answer their clinical questions and to distinguish between trial reports that are likely to be high-quality and contain sufficient data to guide clinical decision-making from those that are not^6^. Within the international physical therapy community, PEDro has been recognized as an important resource to support evidence-based practice for the profession^10^.

Physical therapy as a profession is growing rapidly in Brazil in terms of both the clinical and research workforces. The number of registered physical therapists has nearly doubled in recent years, increasing from 79,382 in 2005^11^ to 152,250 in 2011^12^. Similarly, there was an exponential growth in obtaining postgraduate qualifications^13^. Given this context, as well as the fact that Brazilian scientific physical therapy journals have supported evidence-based practice^14^^-^ 16 and PEDro^14^^,^ 16 ^,^ 17, Brazil might be expected to have high usage of PEDro. An analysis of the usage of PEDro over a 2-year period (2010-2011) showed that Brazil was ranked third for total searches by country worldwide and first among the countries in the South American region of the World Confederation for Physical Therapy (WCPT)^18^. Even though the PEDro search function was only available in English, Brazil ranked higher than many English-speaking countries (including the United Kingdom and Canada). With the exception of the total number of PubMed searches performed each year (total of 10,800 million in 2010-2014^19^), similar usage data for other databases (e.g. Biblioteca Virtual em Saúde^20^), La Biblioteca Cochrane Plus^21^ are not publicly available.

At present, it is unclear whether PEDro is widely and equally used by physical therapists in all parts of Brazil. As PEDro is considered an important resource to support evidence-based physical therapy and makes its usage statistics publicly available, PEDro usage may be considered as an indicator of evidence-based physical therapy. Therefore, the objective of this study was to describe the usage of PEDro in the South American region of the WCPT and, in more detail, in Brazil over a 5-year period (1 January 2010 to 31 December 2014). The specific aims were: (1) to determine the number of PEDro home-page sessions, and (2) to calculate the number of searches performed per year expressed in absolute terms and relative to the number of registered physical therapists and the population size. To characterize these data fully, three units of analysis were considered in the present study: each region of the WCPT, each country in the South American region of the WCPT, and each region in Brazil.

## Method

To quantify usage of PEDro, PEDro home-page^22^sessions were tracked and database search logs were collected over a 5-year period, between 1 January 2010 and 31 December 2014. The 5-year period was chosen as usage data were captured consistently during this time and the most recent complete-year was included.

**Figure 1.**
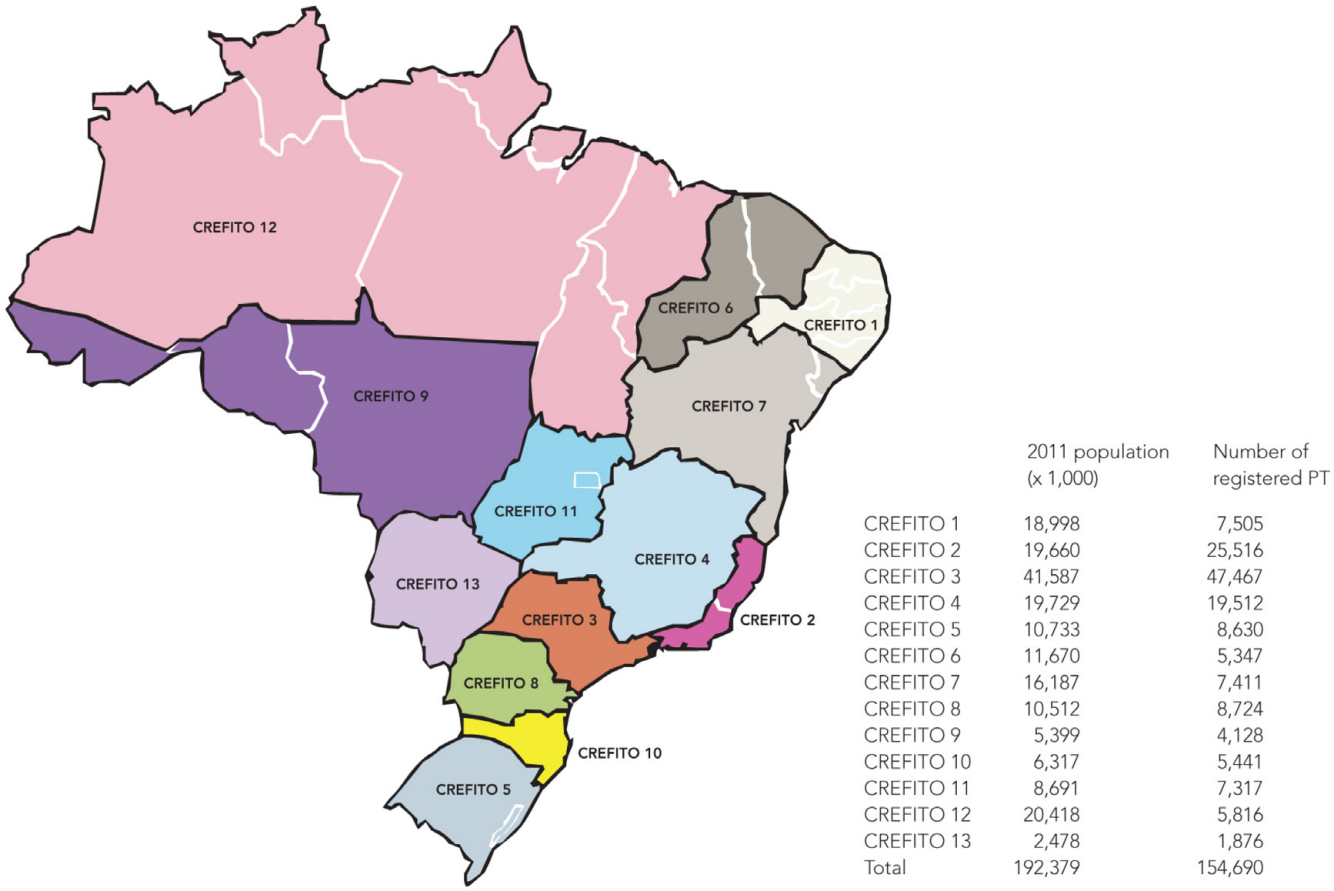
Map of the 26 states and 13 CREFITOs in Brazil.

The WCPT is divided into five regions: Africa, Asia Western Pacific, Europe, North America Caribbean, and South American. Eleven countries (including Brazil) form the South American region. Brazil is divided into 26 states and a federal district, containing the capital city Brasília. In Brazil, the Federal Council of Physical Therapy and Occupational Therapy (Conselho Federal de Fisioterapia e Terapia Ocupacional; COFFITO) regulates and supervises the physical therapy profession at a federal level. Under the COFFITO's supervision, there are 13 Regional Councils, which are responsible for registration of individual occupational and physical therapists at a state or regional level. The Regional Councils are known as the Regional Council of Physical Therapy and Occupational Therapy (Conselho Regional de Fisioterapia e Terapia Ocupacional; CREFITO). Each CREFITO may cover one or more states depending on the number of registered professionals in each state. Figure 1shows the coverage and the number of registered physical therapists in each CREFITO.

Usage of the PEDro home page was determined using data from Google Analytics^23^. Data extraction and analysis have been reported in detail elsewhere^18^. Briefly, the data extracted for the years 2010, 2011, 2012, 2013, and 2014 were: the counts of sessions and the location of each session hosted. The count of sessions represents the number of individual sessions initiated by users of the home page. An individual session occurs when a user opens and navigates around the PEDro home page^23^. The location of each session hosted was derived by mapping internet protocol (IP) addresses^23^. For the analyses of all WCPT regions and countries within the South American region, counts of sessions and locations were grouped respectively at regional and country levels. For the analysis within Brazil, data were extracted separately for each of the 26 states and, then, grouped into the 13 CREFITOs.

The searches performed on the PEDro search function are recorded by the database software, FileMaker Pro 12 (FileMaker Inc., Santa Clara, CA, USA) to July 2014 then MySQL (Oracle Corp, Redwood City, CA, USA) from July 2014, which logs the time and date of each new search performed. To calculate the number of searches performed each year, the date data were exported to an Excel spreadsheet (Microsoft Office 2007, Microsoft Corporation, Redmond, WA, USA). The number of searches from each region, country, and CREFITO was estimated by combining the total number of searches with the proportion of sessions from each region, country, and CREFITO.

Absolute and relative usage of PEDro were calculated for the five WCPT regions, the 11 countries in the South American region of the WCPT, and the 13 CREFITOs in Brazil. Absolute usage was defined as the total number of searches from each region, country, or CREFITO. Relative usage was calculated by dividing the number of searches per year from each region, country, or CREFITO by either the population of the region, country, or CREFITO (number of searches per million-population per year) and/or the number of physical therapists of the region, country, or CREFITO (number of searches per physical therapist per year). Population data for each country were obtained from the United Nations' latest report on world populations^24^. Population data for each state in Brazil were obtained from the Instituto Brasileiro de Geografia e Estatística (IBGE) report for the middle year of our dataset (2011)^25^ and then these data were grouped by CREFITO. While the WCPT is currently conducting a project to calculate the number of physical therapists in each of its Member Organizations^26^, reliable and complete data are unavailable. For this reason, we were only able to calculate the relative usage normalized by the number of registered physical therapists for each CREFITO in Brazil. We used the number of registered physical therapists in each CREFITO for the middle year of our data set (2011)^12^.

## Results

Between 1 January 2010 and 31 December 2014, there were a total of 2,924,691 PEDro home-page sessions globally (397,215 in 2010, 523,966 in 2011, 604,754 in 2012, 745,112 in 2013, and 665,538 in 2014). A total of 7,707,053 searches were performed over the 5-year period (1,577,446 in 2010, 1,773,294 in 2011, 1,773,959 in 2012, 1,155,373 in 2013, and 1,426,981 in 2014).

Table 1 lists the absolute and relative usage of PEDro for each WCPT region, each country in the South American region, and each CREFITO in Brazil. The region with the highest absolute usage was Europe (43.3% of all searches), followed by Asia West Pacific (21.4%) and North America Caribbean (17.2%). When the usage was normalized for the population size this ranking changed to: Europe with 971 searches per million-population per year, followed by North America Caribbean (707) and South American (486).

**Table 1.** Estimated number and percentage of PEDro searches from 2010 to 2014 for each World Confederation for Physical Therapy region, country in South American, and Regional Council (CREFITO) in Brazil.

	Number of searches for 2010-2014	Number of searches per year	% usage	2011 population (x 1,000,000)	Number of registered PT	Number of searches per million-population per year	Number of searches per registered PT per year	Rank by absolute number of searches	Rank by number of search per million‑population per year	Rank by number of searches per registered PT per year
WCPT regions										
Europe	3,335,277	667,055	43.3 %	687.0	-	971	-	1	1	-
North America Caribbean	1,324,138	264,828	17.2 %	374.3	-	707	-	3	2	-
South American	1,240,503	248,101	16.1 %	510.4	-	486	-	4	3	-
Asia Western Pacific	1,648,609	329,722	21.4 %	2,405.7	-	137	-	2	4	-
Africa	68,862	13,772	0.9 %	604.1	-	23	-	6	5	-
No WCPT region	89,664	17,933	1.2 %	2,309.7	-	8	-	5	6	-
*Total*	*7,707,053 *	*1,541,411 *	*100.0 %*	*6,891.3*		*224*				
Countries in WCPT South American region										
Chile	190,776	38,155	15.4 %	17.2	-	2,225	-	2	1	-
Brazil	737,093	147,419	59.4 %	195.2	154,690	755	0.95	1	2	-
Colombia	121,149	24,230	9.8 %	46.4	-	522	-	3	3	-
Peru	29,498	5,900	2.4 %	29.3	-	202	-	6	4	-
Argentina	35,056	7,011	2.8 %	40.4	-	174	-	5	5	-
Mexico	95,124	19,025	7.7 %	117.9	-	161	-	4	6	-
Uruguay	2,098	420	0.2 %	3.4	-	124	-	10	7	-
Ecuador	9,228	1,846	0.7 %	15.0	-	123	-	8	8	-
Bolivia	5,189	1,038	0.4 %	10.2	-	102	-	9	9	-
Venezuela	13,437	2,687	1.1 %	29.0	-	93	-	7	10	-
Paraguay	1,855	371	0.1 %	6.5	-	57	-	11	11	-
*Total*	*1,240,503 *	*248,101 *	*100.0 %*	*510.4*		*486*				
CREFITOs in Brazil										
Crefito 3	264,547	52,909	35.9 %	41.6	47,467	1,272	1.11	1	1	5
Crefito 10	33,884	6,777	4.6 %	6.3	5,441	1,073	1.25	9	2	2
Crefito 4	103,381	20,676	14.0 %	19.7	19,512	1,048	1.06	2	3	7
Crefito 5	48,725	9,745	6.6 %	10.7	8,630	908	1.13	4	4	4
Crefito 8	38,934	7,787	5.3 %	10.5	8,724	741	0.89	7	5	8
Crefito 11	31,178	6,236	4.2 %	8.7	7,317	718	0.85	10	6	9
Crefito 2	69,036	13,807	9.4 %	19.7	25,516	702	0.54	3	7	11
Crefito 6	34,429	6,886	4.7 %	11.7	5,347	590	1.29	8	8	1
Crefito 7	39,416	7,883	5.3%	16.2	7,411	487	1.06	6	9	6
Crefito 1	44,975	8,995	6.1 %	19.0	7,505	473	1.20	5	10	3
Crefito 13	4,588	918	0.6 %	2.5	1,876	370	0.49	13	11	12
Crefito 9	6,440	1,288	0.9 %	5.4	4,128	239	0.31	12	12	13
Crefito 12	17,560	3,512	2.4 %	20.4	5,816	172	0.60	11	13	10
*Total*	*737,093 *	*147,419 *	*100.0 %*	*192.4*	*154,690*	*766*	*0.95*			

Among the countries in the South American region of the WCPT, the three countries with the highest percentage of PEDro searches were Brazil (59.4%), Chile (15.4%), and Colombia (9.8%). When usage was normalized for the population size, Chile (2,225 searches per million-population per year) was ranked first followed by Brazil (755) and Colombia (522). Brazilian physical therapists performed an average of 0.95 PEDro searches per year.

**Figure 2.**
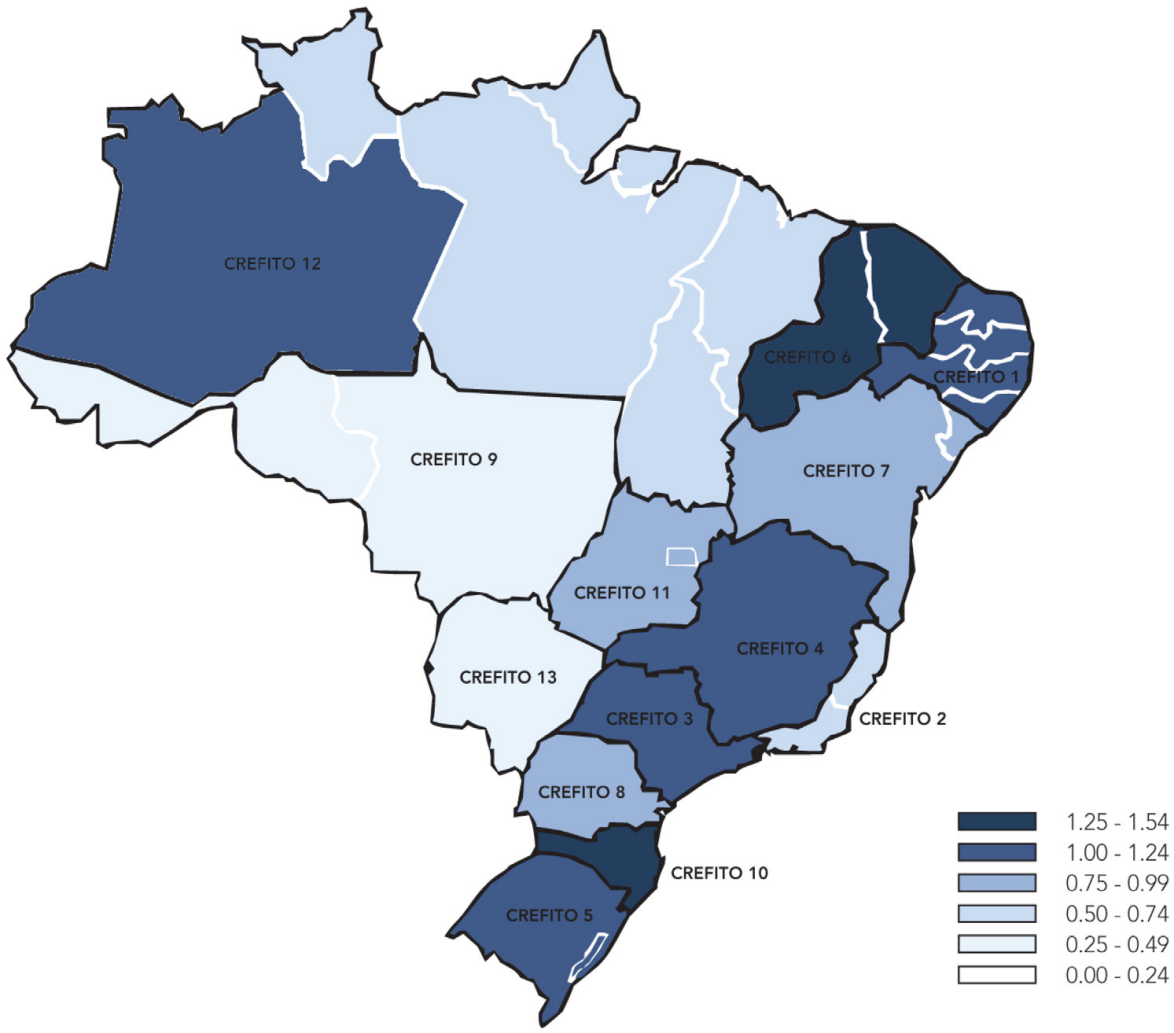
PEDro usage relative to the number of registered physical therapists in each CREFITO in Brazil (i.e. number of searches per year per physical therapist). Darker shading represents higher usage.

Usage at the national level showed that the three CREFITOs with the highest number of PEDro searches in absolute terms were all from the southeast region of Brazil: CREFITO 3 (35.9%; São Paulo), CREFITO 4 (14.0%; Minas Gerais) and CREFITO 2 (9.4%; Espírito Santo and Rio de Janeiro). CREFITO 3 and 4 retained their top rankings when the data were normalized for the population size, with 1,272 (ranked first) and 1,048 (ranked third) searches per million-population per year. CREFITO 10 (Santa Catarina) was ranked second with 1,073 searches per million-population per year. However, when the data were normalized for the number of registered physical therapists, CREFITO 6 (Ceará and Piauí) ranked first with 1.29 searches per physical therapist per year, followed by CREFITOs 10 and 1 (Alagoas, Paraíba, Pernambuco, and Rio Grande do Norte) with 1.25 and 1.20 searches per physical therapist per year each, respectively. CREFITOs 13 (Mato Grosso do Sul), 12 (Amapá, Amazonas, Maranhão, Pará, Roraima, and Tocantins) and 9 (Acre, Mato Grosso, and Rondônia) were ranked the lowest in both absolute and relative usage. Figure 2 shows the results for the CREFITO analysis.

## Discussion

Europe had the highest absolute and relative usage of PEDro across the five WCPT regions, with the South American region ranked third in relative terms and fourth in absolute terms. Within the South American region, Brazil accounted for the highest absolute usage (nearly 60% of searches) and was second after Chile for relative usage. PEDro usage in Brazil is not equally distributed across the CREFITOs. Brazilian physical therapists performed, on average, less than one PEDro search per year. This finding raises the issue of how well PEDro and evidence-based practice have been promoted and disseminated among Brazilian physical therapists.

The high PEDro usage in Europe is not surprising given that a previous study, which analyzed a subset of the data presented in this study, found eight European countries among the twelve countries with the highest number of searches^18^. Europe is the region with the highest number of members affiliated to the WCPT, which has been supporting dissemination and implementation of evidence-based practice for more than a decade^27^. A limitation of our analysis is that we could not calculate usage normalized by the number of physical therapists for countries and regions outside Brazil because data on the number of physical therapists in each country are not easily accessible. The WCPT is currently establishing a common data set (including the number of registered and unregistered physical therapists practicing in each country) that will address this limitation in our analysis^26^.

PEDro usage has not been widely and equally disseminated at a national level in Brazil. For instance, low relative and absolute usages were more evident among CREFITOs located in the North and Central-West regions of Brazil (CREFITO 13, 12, and 9). CREFITOs from other regions, such as CREFITO 6, 10, and 1, performed slightly better when usage was normalized by the number of physical therapists. However, our results showed that, overall, Brazilian physical therapists performed less than one search per year on PEDro. The best estimate was from CREFITO 6 with 1.29 searches per physical therapist per year. These findings suggest that a greater effort is needed to promote PEDro and evidence-based practice within the Brazilian physical therapy community. Dissemination of the concept of evidence-based practice often starts at the university level, and then this practice should ideally continue to be pursued by physical therapists throughout their career. In Brazil, strategies to promote evidence-based competencies (including using PEDro to search for high-quality research to answer clinical questions about the effects of physical therapy interventions) targeting students at an undergraduate or postgraduate level seem to be a reasonable approach. The number of physical therapists is likely to continue to increase in the near future, as there are currently 620 physical therapy undergraduate courses active in Brazil^28^. Integration of evidence-based practice and information literacy early into undergraduate programs is effective^29^, but the challenge is to sustain the acquired knowledge beyond the early professional working years^30^.

One limitation of our study is that Brazilian physical therapists might also use other online resources to search for evidence rather than searching PEDro exclusively, such as PubMed^31^ and *Portal de Evidências* from *Biblioteca Virtual em Saúde* 20 and La Biblioteca Cochrane Plus from Centro Cochrane Iberoamericano^21^. We were unable to locate usage statistics for databases other than PEDro, so it is not possible to confirm this supposition nor to compare PEDro usage to other databases in different countries or regions. Given that the PEDro resource is specific to physical therapy and indexes guidelines, reviews, and trials about the effects of physical therapy interventions only, it is not surprising that the number of PEDro searches performed in 2010-2014 (7.7 million) was significantly lower than the number of PubMed searches for the same period (10,800 million^19^).

One barrier to the widespread usage of PEDro in Brazil is that the search function is only available in English. While the PEDro home page is available in 10 languages, including Portuguese, the search function in English can pose an obstacle to physical therapists whose first language is not English. The *Portal de Evidências* is a trilingual interface (Portuguese, English, and Spanish) that allows simultaneous search in multiple databases (including The Cochrane Library and Lilacs). Searching in non-English languages, like the *Portal de Evidências,* could be implemented in PEDro. However, this would require a significant investment of money by the global physical therapy community because of the costs involved in translation. Alternatively, evaluation of emerging technologies like automated translation of webpages and exploring partnerships with existing multilingual evidence portals (e.g. *Biblioteca Virtual em Saúde*) are warranted.

A strength of our study was the use of numbers of registered physical therapists to calculate relative usage per CREFITO. However, the data used in this study were not readily available from the COFFITO and only half of the CREFITOs make this data available on their website. Instead, our estimates were derived from another source^12^. Although we are confident in these figures, for transparency and research purposes, we would advocate that this type of statistic should be freely available in the COFFITO and CREFITOs websites. A weakness of this study is that we could not delineate who was using PEDro (i.e. physical therapists, other health professionals, or consumers) nor whether the users were primarily clinicians, educators, or researchers. Factors such as the ratio between clinical and research physical therapists as well as internet accessibility in each area, country, and region could also affect PEDro usage.

In summary, our findings revealed that, although Brazil has a higher PEDro usage than other South American countries, usage normalized by number of physical therapists is very low across Brazil. Strategies to promote PEDro effectively to Brazilian physical therapists are needed. Future surveys of Brazilian physical therapists might help to understand the factors associated with PEDro usage behaviors and identify targets to promote the uptake of evidence-based practice more effectively across the nation.

## References

[1] Nilsen P, Bernhardsson S (2013). Towards evidence-based physiotherapy - research challenges and
needs. J Physiother.

[2] Herbert R, Jamtvedt G, Hagen KB, Mead J (2011). Practical evidence-based physiotherapy.

[3] Sackett DL, Straus SE, Richardson WS, Rosenberg W, Haynes RB (2000). Evidence-based medicine: how to practice and teach EBM.

[4] Scurlock-Evans L, Upton P, Upton D (2014). Evidence-based practice in physiotherapy: a systematic review of
barriers, enablers and interventions. Physiotherapy.

[5] Oxford Centre for Evidence-Based Medicine (2011). The Oxford 2011 levels of evidence. [Internet].

[6] Sherrington C, Herbert RD, Maher CG, Moseley AM. PEDro (2000). A database of randomized trials and systematic reviews in
physiotherapy. Man Ther.

[7] Centre for Evidence-Based Physiotherapy (2015). PEDro statistics.

[8] Michaleff ZA, Costa LOP, Moseley AM, Maher CG, Elkins MR, Herbert RD (2011). CENTRAL, PEDro, PubMed, and EMBASE are the most comprehensive
databases indexing randomized controlled trials of physical therapy
interventions. Phys Ther.

[9] Moseley AM, Sherrington C, Elkins MR, Herbert RD, Maher CG (2009). Indexing of randomised controlled trials of physiotherapy
interventions: a comparison of AMED, CENTRAL, CINAHL, EMBASE, hooked on evidence,
PEDro, PsycINFO and PubMed. Physiotherapy.

[10] World Confederation for Physical Therapy (2014). Professional partnerships [Internet].

[11] Andrade A, Lemos J, Dall'Ago P, Haddad AE (2006). Fisioterapia. A trajetória dos cursos de graduação na saúde: 1991-2004.

[12] Oliveira GCC (2011). Emergência de realidades no ensino superior da saúde: atos e vozes da área de
fisioterapia nas diretrizes curriculares nacionais.

[13] Coury HJCG, Vilella I (2009). Perfil do pesquisador fisioterapeuta brasileiro. Rev Bras Fisioter..

[14] Dias RC, Dias JMD (2006). Evidence-based practice: a methodology for a best physical therapy
practice. Fisioter Mov.

[15] Filippin LI, Wagner MB (2008). Fisioterapia baseada em evidência: uma nova
perspectiva. Rev Bras Fisioter.

[16] Marques AP, Peccin MS (2005). Research in physical therapy: the evidence grounded practice and study
models. Fisioter Pesqui.

[17] Shiwa SR, Costa LOP, Moser ADL, Aguiar IC, Oliveira LVF (2011). PEDro: a base de dados de evidências em fisioterapia. Fisioter Mov..

[18] Campos TF, Beckenkamp PR, Moseley AM. (2013). Usage evaluation of a resource to support evidence-based
physiotherapy: the Physiotherapy Evidence Database (PEDro). Physiotherapy.

[19] U. S. National Library of Medicine (2015). Key MEDLINE(r) Indicators [Internet].

[20] (2015). BIREME. Biblioteca Virtual em Saúde.

[21] Centro Cochrane Iberoamericano (2015). La Biblioteca Cochrane Plus.

[22] Centre for Evidence-Based Physiotherapy (2015). Physiotherapy Evidence Database.

[23] Google (2015). Google analytics.

[24] United Nations Population Division,Department of Economic and Social
Affairs (2013). World population prospects: the 2012 revision, file POP/1-1: total population
(both sexes combined) by major area, region and country, annually for 1950-2100
(thousands), estimates 1950-2010 [Internet].

[25] Instituto Brasileiro de Geografia e Estatística - IBGE (2015). Estimativas de população 2011.

[26] World Confederation for Physical Therapy (2013). Data collection project - data guide for 2013 [Internet].

[27] World Confederation for Physical Therapy (2011). Policy statement - evidence based practice [Internet].

[28] Brasil.Ministério da Educação (2015). Sistema e-MEC. Instituições de Educação Superior e Cursos
Cadastrados.

[29] Boruff JT, Thomas A. (2011). Integrating evidence-based practice and information literacy skills in
teaching physical and occupational therapy students. Health Info Libr J..

[30] McEvoy MP, Williams MT, Olds TS, Lewis LK, Petkov J (2011). Evidence-based practice profiles of physiotherapists transitioning
into the workforce: a study of two cohorts. BMC Med Educ..

[31] National Center for Biotechnology Information, U.S (2015). National Library of Medicine.

